# Beyond inpatient and outpatient care: alternative model for hypertension management

**DOI:** 10.1186/1471-2458-6-257

**Published:** 2006-10-19

**Authors:** P Michael Ho, John S Rumsfeld

**Affiliations:** 1 Cardiology Section, Denver VA Medical Center, 1055 Clermont Street (111B), Denver, Colorado 80220, USA

## Abstract

Hypertension is a major contributor to worldwide cardiovascular mortality, however, only one-third of patients with hypertension have their blood pressure treated to guideline recommended levels. To improve hypertension control, there may need to be a fundamental shift in care delivery, one that is population-based and simultaneously addresses patient, provider and system barriers. One potential approach is home-based disease management, based on the triad of home monitoring, team care, and patient self-care. Although there may be challenges to achieving the vision of home-based disease management, there are tremendous potential benefits of such an approach for reducing the global burden of cardiovascular disease.

## Text

Hypertension is a principal risk factor for the development of coronary heart disease and stroke, and is a major contributor to worldwide cardiovascular mortality [1]. It affects up to 37% of the global adult population and it is estimated that 7.1 million deaths are due to hypertension, which is 13% of total global fatality [1]. The continued worldwide burden of hypertension is surprising given awareness of the importance of blood pressure (BP) control by public health agencies, the medical community and the public, coupled with the availability of safe and effective therapies. Currently, there are over 10 classes of anti-hypertensive medications and it is estimated that achieving a sustained 12 mm Hg decrease in systolic BP will prevent 1 death for every 11 patients treated [2]. Despite these known benefits, only one-third of patients with hypertension have their blood pressure treated to guideline recommended levels [2].

Improved hypertension management thereby remains a primary global public health goal, but how to best achieve broad hypertension control remains uncertain. The study by Heinz et al. adds to our knowledge about potential approaches to the management of blood pressure [3]. The study found that, among a high-risk cohort of hypertensive patients with left ventricular hypertrophy in Germany, 68% of patients had their BP controlled after an intensive inpatient rehabilitation stay while 45% of outpatients had their BP controlled after a mean follow-up of 52 days.

Superficially, one is tempted to conclude that there may be a role for initial inpatient management of hypertension to ensure better chronic BP control. However, the study by Heinz et al. does not support such a conclusion. In this observational study, patients who received inpatient BP management had significantly lower BP from the start of the observation period, and the proportional reductions in BP were equal between the two groups over the follow-up period. Thus, the authors correctly concluded that the principal finding of this study were: 1) patients who had inpatient BP management, despite more prevalent cardiovascular disease and comorbidites, had lower BP than those who had outpatient management from the outset; and 2) there was, "a high proportion of patients that did not achieve treatment goals" in both groups [3]. While the rates of BP control in this study were higher than those reported in the literature, 32% of the inpatient group and 55% of the outpatient group were left with uncontrolled hypertension.

This begs the question of why hypertension management is so difficult. The answer may lie in the fact that BP control is related to patient, provider and healthcare system factors, all of which must align to achieve BP goals. From a patient perspective, hypertension is often a silent disease and patients may not take antihypertensive medications as directed because their positive effects are not as obvious as potential side effects from the medications. Moreover, patients with hypertension often have co-morbid diseases (e.g., diabetes) that require additional medications, further increasing the complexity of medication regimens. Patients may also have conditions such as depression that directly impact adherence to therapy [4]. From a provider perspective, potential explanations for difficulty controlling hypertension include therapeutic inertia, where providers fail to intensify therapy despite persistently elevated BP readings, and the 'tyranny of the urgent', where office visits are focused on acute complaints, the need to address multiple chronic conditions, and administrative work such as medication refills, leaving hypertension management a lower priority [5,6]. Finally, multiple system-level issues can negatively impact hypertension management. Examples of these include lack of access to care, reliance on episodic patient-provider visits, care delivered in silos (e.g. specialist versus generalist), and failure to engage patients in their own management.

Small studies of quality improvement (QI) interventions for hypertension have addressed some of these barriers and have achieved modest results [7]. Multi-modal interventions have been the most successful with the following general hierarchy of effectiveness: team management, patient education, and provider-centered interventions [7]. Team management has generally consisted of assigning patient care responsibilities to someone other than the patient's physician, with this person also taking responsibility for patient education and follow-up. Overall, patients in the intervention groups achieved median reductions of 4.5 mm Hg for systolic and 2.1 mm Hg for diastolic blood pressures [7]. Despite the efficacy of some hypertension interventions, they can be resource intensive and their broad applicability and effectiveness in clinical practice are unclear. Certainly, they have not been widely adopted and the gaps in BP control persist.

To improve hypertension management, there may need to be a fundamental shift in care delivery, one that is population-based and simultaneously addresses patient, provider and system barriers. One potential approach is to shift from reliance on traditional, episodic visits to home-based disease management for all patients with hypertension. To achieve this, health information technology would need to be employed for chronic home monitoring and management (e.g. interactive voice response technology, home telemonitoring devices, or the Internet), drastically reducing the need for office visits by patients. Then, teams comprised of pharmacists, nurses, nurse practitioners, and/or physician assistants, with physician oversight, would make management decisions based on home monitoring and remote patient communication. Of note, this creates an efficiency whereby a team can remotely manage many more patients than can possibly be done in the office setting. In addition, intensity of care can be tailored depending on the clinical need, with frequent management interventions to achieve blood pressure control in the initial phase, and lower levels of surveillance later on for patients who have achieved BP goals and are feeling well. As important, patients would practice self-care (e.g. home blood pressure measurement, medication adherence, daily weights, reporting of exercise and diet) and receive education about hypertension management and lifestyle modifications through the home monitoring technology. Office visits for hypertension would be used to complement home-based management and for clinical situations where a face-to-face visit is required.

Taking this a step further, hypertension is usually one of a number of conditions that contribute to a patient's overall risk for cardiovascular disease. To address the growing number of patients with multiple risk factors, there needs to be a shift away from focusing on single disease conditions towards global cardiovascular disease risk assessment and reduction. The majority of patients with hypertension often have co-existing conditions such as diabetes, high cholesterol, smoking, and/or sedentary lifestyles that require chronic ongoing care and these conditions may also benefit from the triad of home monitoring, team care, and patient self-care. Rather than treating hypertension as an isolated condition, the home-based disease management program can be tailored to a patient's cardiovascular disease risk profile and treatment intensity can be titrated accordingly.

There are obvious challenges to achieving the vision of home-based disease management. For example, physicians would need to embrace health information technology and team management as principal methods of routine patient care. Moreover, health care policies must align financial incentives to support such programs, including the infrastructure to support the technology and appropriate reimbursement for utilizing such systems to provide chronic care management. However, the gains of pursuing such a vision may be tremendous, including 1) efficiencies of management, 2) potential for broad application for populations of patients and across multiple chronic conditions, 3) early detection of patient problems/decompensation at home, 4) allowing physicians to concentrate on sicker patients, acute conditions, and diagnostic workups in the office, 5) ensuring continuity of care, and 6) directly activating patients to engage in self-care.

Perhaps the most compelling reason to push for a shift to home-based disease management is that current care models are clearly insufficient. The study by Heinz et al. tells us that even intensive inpatient management doesn't get the job done. The growing pressures on clinicians within office and hospital settings are likely to relegate hypertension management even lower on the priority list. At the same time, disease management trials for heart failure support the idea that home monitoring coupled with team care and patient self-care can improve patient outcomes [8]. It is notable that healthcare systems with aligned incentives to take responsibility for 'covered lives' rather than episodes of care, like Kaiser Permanente and the Veterans Health Administration, are rapidly moving to such chronic disease management programs for their patients. Over 16 million people die worldwide each year from cardiovascular disease [9]. If we are to make an impact in reducing the global burden of cardiovascular disease, the time for change is now and home-based chronic disease management offers an alternative model of care with tremendous potential benefits.

## Pre-publication history

The pre-publication history for this paper can be accessed here:


